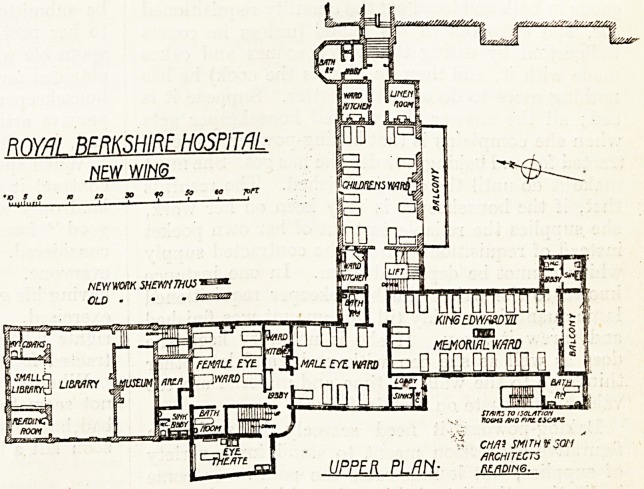# The Alterations at the Royal Berkshire Hospital

**Published:** 1912-10-26

**Authors:** 


### HOSPITAL ARCHITECTURE AND CONSTRUCTION.

[Communications on this subject should be marked "Architecture" in the left-hand top corner of the envelope.]

### The Alterations at the Royal Berkshire Hospital.

A new wing and extensive alterations and additions to the out-patient department have been carried out at this hospital from designs by Messrs. Charles Smith and Son, architects, of Reading. These works are illustrated by plans of the two principal floors in which the new work is shown in black, the old work being indicated by diagonal hatching.

GROUND FLOOR PLAN

In the out-patient department the main waiting hall has been enlarged by the addition of about 50 per cent. more floor space, and both dressing rooms adjoining the surgeon's consulting room have been extended. For the physician a complete new suite of three rooms has been erected; the dispensary, together with store and dining room for dispensers, is completely new, as also are the sanitary offices for out-patients.

The access from the waiting hall to the physician's rooms is not conveniently arranged, but probably the architects were hampered here by the necessity for preserving the staircase to the library and museum on the floor above. There is no separate waiting room for medicine, and the arrangements for the exit of patients is not quite clear from the plan, but it would seem that some amount of crossing is inevitable. An open archway separates the out-patient department from the casualty department, which is, except the large waiting hall, entirely new. The accommodation comprises a porter's office, *x*-ray room, receiving room, surgery, dressing room, surgeon's room, and operation room.

On the first floor, partly over the out-patient department and partly over the casualty department, are two wards for male and female eye patients respectively. These wards are approached by two staircases, one close to the dispensary and the other between the new building and the old. The eye wards afford accommodation for eight male and six female patients, and a ward kitchen; the usual sanitary offices and an operation theatre are provided. The ventilation of the female

UPPER PLAN

eye ward is not all that could be wished. There are two windows on the east wall giving on to the open space in front of the hospital and one on the north opening into a small internal area; the fireplace being on the east side between the two windows referred to. The bath room attached to the female ward would appear from the plan to have neither light nor ventilation. The remainder of the space over the casualty department is occupied by a new ward—"The King Edward VII. Memorial Ward." This ward contains twenty beds for children, and is provided with a bath room and the usual sanitary offices. Access is arranged from the bath room to the outside escape staircase, which is also the way up to the isolation wards.

In planning the ward the obsolete system of coupled beds has been adopted; and part of the ward containing five beds is devoid of any attempt at cross-ventilation. A little rearrangement and some extension southward, even if the motor shed had to be sacrificed, would have obviated what we cannot but regard as an unfortunate blunder. On the floor above are nurses' bed and sitting rooms.

In the south-east angle of the site a new pathological block has been erected comprising a mortuary chapel and post-mortem room.

## Figures and Tables

**Figure f1:**
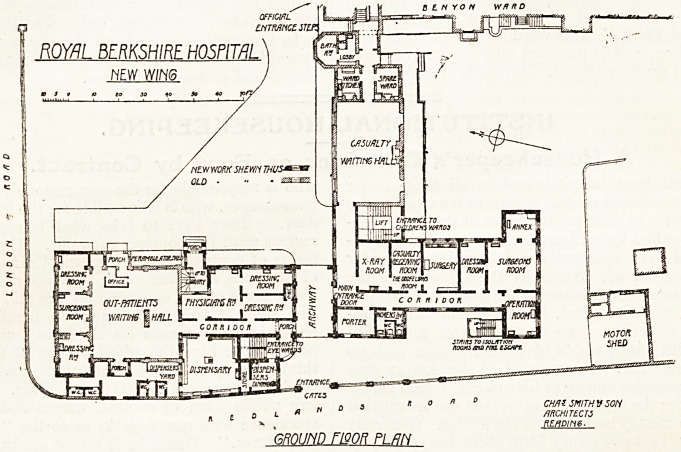


**Figure f2:**